# How does poverty affect children’s nutritional status in Nairobi slums? A qualitative study of the root causes of undernutrition

**DOI:** 10.1017/S1368980016002445

**Published:** 2016-09-20

**Authors:** Sophie M Goudet, Elizabeth W Kimani-Murage, Frederick Wekesah, Milka Wanjohi, Paula L Griffiths, Barry Bogin, Nyovani J Madise

**Affiliations:** 1 School of Sport, Exercise & Health Sciences, Loughborough University, Epinal Way, Loughborough, Leicestershire, LE11 3TU, UK; 2 African Population and Health Research Center (APHRC), Research Nairobi, Nairobi, Kenya; 3 Julius Global Health, Julius Center for Health Sciences and Primary Care, Utrecht Medical Center, Utrecht, The Netherlands; 4 Centre for Global Health, Population, Poverty & Policy, University of Southampton, Southampton, UK

**Keywords:** Root cause, Infant and young child, Undernutrition, Slums, Nairobi

## Abstract

**Objective:**

Children in slums are at high risk of undernutrition, which has long-term negative consequences on their physical growth and cognitive development. Severe undernutrition can lead to the child’s death. The present paper aimed to understand the causes of undernutrition in children as perceived by various groups of community members in Nairobi slums, Kenya.

**Design:**

Analysis of ten focus group discussions and ten individual interviews with key informants. The main topic discussed was the root causes of child undernutrition in the slums. The focus group discussions and key informant interviews were recorded and transcribed verbatim. The transcripts were coded in NVivo by extracting concepts and using a constant comparison of data across the different categories of respondents to draw out themes to enable a thematic analysis.

**Setting:**

Two slum communities in Nairobi, Kenya.

**Subjects:**

Women of childbearing age, community health workers, elders, leaders and other knowledgeable people in the two slum communities (*n* 90).

**Results:**

Participants demonstrated an understanding of undernutrition in children.

**Conclusions:**

Findings inform target criteria at community and household level that can be used to identify children at risk of undernutrition. To tackle the immediate and underlying causes of undernutrition, interventions recommended should aim to: (i) improve maternal health and nutrition; (ii) promote optimal infant and young children feeding practices; (iii) support mothers in their working role; (iv) increase access to family planning; (v) improve water, sanitation and hygiene (WASH); (vi) address alcohol problems at all levels; and (vii) address street food issues with infant feeding counselling.

By 2030, urban slum populations of less developed countries are expected to double to almost two billion people^(^ ^1^ ^)^. In often appalling conditions, slum-dwellers frequently lack access to safe drinking-water, sanitation, security of tender, durable housing and access to proper health care^(^ ^1^ ^,^ ^2^ ^)^. These elements, combined with overcrowding, mean that slum-dwellers are exposed to an increased risk of infectious diseases^(^ ^2^ ^)^. Women, infants and young children are typically the ones who suffer the most, resulting in poor nutritional health, high mortality/morbidity rates and a high risk of intergenerational malnutrition^(^ ^3^ ^–^ ^18^ ^)^. Malnourished children are likely to have impaired cognitive, intellectual and physical development, with permanent consequences in their adult life. Malnutrition not only impacts on human development but also on national economic growth^(^ ^19^ ^,^ ^20^ ^)^.

In Nairobi, Kenya, the estimated number of slum dwellers is approximately one million inhabitants^(^ ^21^ ^)^ or 32 % of the total population, who occupy approximately 6 % of Nairobi’s residential land^(^ ^22^ ^)^. The slums in Nairobi have poor housing, no potable water and waste disposal, and are characterized by high poverty, high levels of violence and insecurity, unemployment and poor health indicators^(^ ^23^ ^–^ ^25^ ^)^. Children in the slums have about 12 % higher mortality and morbidity rates than children in other parts of Kenya and other urban residents^(^ ^26^ ^–^ ^29^ ^)^. They also have low health-care utilisation and are less likely to be vaccinated^(^ ^30^ ^)^. The nutritional status of children in Nairobi slums is poor. Stunting, which results from chronic undernourishment and repeated exposure to morbidity, is high, between 33·5 %^(^ ^31^ ^)^ and up to 47 %^(^ ^32^ ^,^ ^33^ ^)^ *v*. 19·8 % in other urban areas^(^ ^34^ ^)^. Wasting, representative of acute malnutrition, is respectively 3·5 % for global acute malnutrition and 0·3 % for severe acute malnutrition^(^ ^31^ ^)^. Although these are under the threshold of a serious situation (global acute malnutrition >10 %), these represent a huge caseload of malnourished children (approximately 40 000 children, based on the estimated number of children in Nairobi slums <5 years old × global acute malnutrition rate of 3·5 %). Poor infant feeding practices have been identified in the Nairobi slums. Exclusive breast-feeding for 6 months is barely 2 %^(^ ^35^ ^,^ ^36^ ^)^. Complementary feeding practices are suboptimal especially with regard to the nutrient density of the foods fed to children under 2 years old^(^ ^36^ ^)^. Child-level factors such as child’s sex and perceived size at birth and maternal characteristics including marital status, ethnicity and education level are associated with suboptimal feeding practices^(^ ^35^ ^,^ ^37^ ^)^.

The root causes of malnutrition in children are conceptualised in the UNICEF conceptual framework^(^ ^38^ ^)^. This framework classifies the root causes as immediate (individual level, such as sickness), underlying (household or family level, such as food insecurity) and basic (societal level, such as the place of women in the society). This framework is not context-specific and does not reflect the urban setting specificities (e.g. what are the prevalent diseases causing undernutrition in slums?) and the risk factors associated with a root cause (e.g. what is causing inadequate dietary intake in slums?). These are relevant questions as previous research has shown that urban slum conditions and environment impact children’s nutritional health^(^ ^16^ ^,^ ^39^ ^–^ ^42^ ^)^ and that the determinants of undernutrition at the individual, household, community and country level in an urban environment have an independent effect on children’s health and nutritional status^(^ ^39^ ^,^ ^42^ ^–^ ^46^ ^)^.

In the present study, our aim was to explore community members’ views on the causes of undernutrition in children in urban poor settings in Nairobi, Kenya and to identify locally conceptualised pathways leading to undernutrition. It is important to understand the causes of undernutrition as perceived by various community members, to complement and deepen the understanding of quantitative findings on the causes of undernutrition. The knowledge of community members on the causes of children’s undernutrition and their associated risk factors can serve as baseline information for nutrition education interventions. The analysis can also identify differences in perceptions that can be used to target specific groups within the slum communities to promote child’s health. This new understanding can inform targeting criteria for children at risk of undernutrition as well as the design of context-specific interventions to promote better health among children. Finally, a better understanding of how the victims of child undernutrition perceive the problem may help to change public and private policies that deprive residents of urban slums their basic human needs.

## Methods

### Study setting and population

The study was conducted in two slums of Nairobi, Kenya, namely Korogocho and Viwandani, which are included in the Nairobi Urban Health and Demographic Surveillance System^(^ ^47^ ^)^. The two slums are located about 7 km from each other, occupy a total area of slightly less than 1 km^2^ and are densely populated (average 57 950 inhabitants/km^2^). Viwandani, being located in the industrial area, attracts migrant workers especially men with relatively higher levels of education. Korogocho has a more stable population and greater co-residence of spouses but higher unemployment levels^(^ ^47^ ^)^.

### Data collection

The data used here were part of a formative study conducted in April 2012. The formative study aimed to understand infant and young child feeding (IYCF) practices, the community’s perceptions of the root causes of child undernutrition, and the local contexts and norms which contribute towards decision making for IYCF practices. The findings from the formative study on IYCF practices informed the design of an intervention for which details are published elsewhere^(^ ^48^ ^)^. The intervention involved home-based counselling of pregnant women and mothers of young children on optimal maternal, infant and young child nutrition by community health workers.

We have previously identified factors affecting actualisation of the WHO breast-feeding recommendations from this work^(^ ^37^ ^)^. Here we use the data related to understanding the root causes of undernutrition with the question posed ‘Why do you think children grow undernourished in your community?’ Participants were recruited through purposive sampling depending on category of respondents, taking into account different ethnicity, religious affiliation and village of residence. Interviews were recorded and transcribed verbatim.

The present paper draws on the analysis of ten focus group discussions (FGD) and ten individual interviews with key informants (KII). Both the FGD and the KII were conducted with women of childbearing age, community health workers, community elders, community leaders and other knowledgeable people in the two slum communities (Tables 1 and 2). Two KII were conducted with each participant profile (health-care provider, religious leader, traditional birth attendant, youth leader, women’s group leader), of which 40 % were men and 50 % were in Korogocho. Three FGD were conducted with young mothers, three with older mothers (≥25 years old), two with community health workers and another three with community elders. Pictures of children depicting different nutritional statuses and of foods were used to stimulate responses from respondents. Questions included perceptions on the nutritional status of the majority of infants living in the local community; knowledge, attitudes and practices with regard to maternal, infant and young child nutrition including initiation of breast-feeding, use of colostrum, exclusive breast-feeding, duration of breast-feeding and complementary feeding; and nutritional status of children growing in urban slums. Additionally, questions focused on the contextual and sociocultural norms that influence IYCF practices. Interviews were conducted by ten experienced field interviewers (seven females and three males) with university training in nutrition, public health, sociology or anthropology. The field interviewers were trained to ensure they understood the concepts and the meanings of the questions and on how to engage the participants to ensure frank and complete responses. Role plays among the interviewers and pilot field interviews were conducted as part of the training sessions to ensure the interviewers grasped the concepts. Some of the researchers accompanied the field team in pilot interviews. Debriefing sessions between the interviewers and researchers were held after the pilot interviews to discuss emerging issues and to ensure consistency of meaning to questions. There was always an interviewer/moderator and a note-taker in each interview to ensure all issues discussed were captured. Interviews were conducted in Swahili, and all were audio-taped and transcribed verbatim. Concurrent transcription and translation was done by two graduates with good experience in anthropology and transcription who had participated in the training of the interviewers and the pilot sessions.

**Table 1:** Details of participants in the key informant interviews (KII) conducted in two slum communities in Nairobi, Kenya, April 2012

KII no.	Profile	Age (years)	Gender	Slum
1	Health-care provider	26	M	Korogocho
2	Religious leader	65	F	Korogocho
3	Traditional birth attendant	35	F	Korogocho
4	Women’s group leader	32	F	Korogocho
5	Youth leader	32	M	Korogocho
6	Health-care provider	26	F	Viwandani
7	Religious leader	47	M	Viwandani
8	Women’s group leader	64	F	Viwandani
9	Traditional birth attendant	60	F	Viwandani
10	Youth leader	25	M	Viwandani

M, male; F, female.

**Table 2:** Details of participants in the focus group discussions (FGD) conducted in two slum communities in Nairobi, Kenya, April 2012

FGD no.	Participants (*n*)	Profile	Average age (years)	Gender	Slum
1	8	Community health workers	32	M (*n* 1)	Korogocho
				F (*n* 7)	
2	9	Young mothers	22	F	Korogocho
3	9	Young mothers	21	F	Korogocho
4	6	Older mothers	32	F	Viwandani
5	10	Community elders	44	M (*n* 8)	Viwandani
				F (*n* 2)	
6	7	Young mothers	20	F	Viwandani
7	10	Older mothers	33	F	Korogocho
8	7	Older mothers	29	F	Korogocho
9	7	Community elders	50	M (*n* 5)	Korogocho
				F (*n* 2)	
10	7	Community health workers	50	M (*n* 2)	Viwandani
				F (n 5)	

M, male; F, female.

### Data analysis

Word transcripts were imported into NVivo 10 software (QSR International Pty Ltd) which helped to identify primary and meta codes and major themes. They were coded by extracting concepts and using a constant comparison of the data across different participant groups to identify similarities and variations and to draw out themes based on the UNICEF conceptual framework^(^ ^49^ ^,^ ^50^ ^)^. Themes were developed from the literature and from the narratives from the respondents. The researchers familiarised themselves with the data by listening to the audio tapes and reading the transcripts. Coding and interpretation were done by two members of the research team to ensure objectivity and to check for consistency in application of the coding process. Final checks for understanding and consistency of the application of the codes were undertaken with a third member of the research team.

### Ethical considerations

Ethics approval was granted by the Kenya Medical Research Institute, a recognised Ethics Review Committee, approved by the Government of Kenya. The investigators respected the fundamental principles regarding research on human subjects. For all data collection activities, informed consent was sought from the eligible participants following full disclosure regarding the study before data collection was done.

## Results

There were a total of ninety individuals participating in FGD (*n* 80) and KII (*n* 10). Participants’ sociodemographic characteristics are presented in Table 3.

**Table 3:** Sociodemographic characteristics of participants in the key informant interviews and focus group discussions conducted in two slum communities in Nairobi, Kenya, April 2012

Sociodemographic characteristic	Men	Women	Total
Age			
Mean age (years)	41·8	30·5	–
<25 years (*n*)	0	27	27
≥25 years (*n*)	20	44	64
Religion (*n*)			
Christian	19	59	78
Muslim	1	12	13
Missing	0	0	0
Ethnic background (*n*)			
Kikuyu	2	19	21
Kamba	5	14	19
Luo	4	19	23
Luhya	4	6	10
Somali/Borana	0	9	9
Other	5	4	9
Education status (*n*)			
None	0	4	4
Pre-primary	3	6	9
Primary	5	42	47
Secondary	7	17	24
Post-secondary	5	2	7
Occupation (*n*)			
Casual worker	10	4	14
Community health/social worker	3	11	14
Employed	1	2	3
Business/self-employed	7	16	23
Community leaders	1	5	6
Unemployed	0	28	28
Student	0	2	2
Missing	0	1	1
Marital status (*n*)			
Married	18	40	58
Widowed	0	3	3
Single/not married	2	28	30
Missing	0	0	0
Slum (*n*)			
Korogocho	8	48	56
Viwandani	12	23	35

### Why do children become undernourished?

**Fig. 1:**
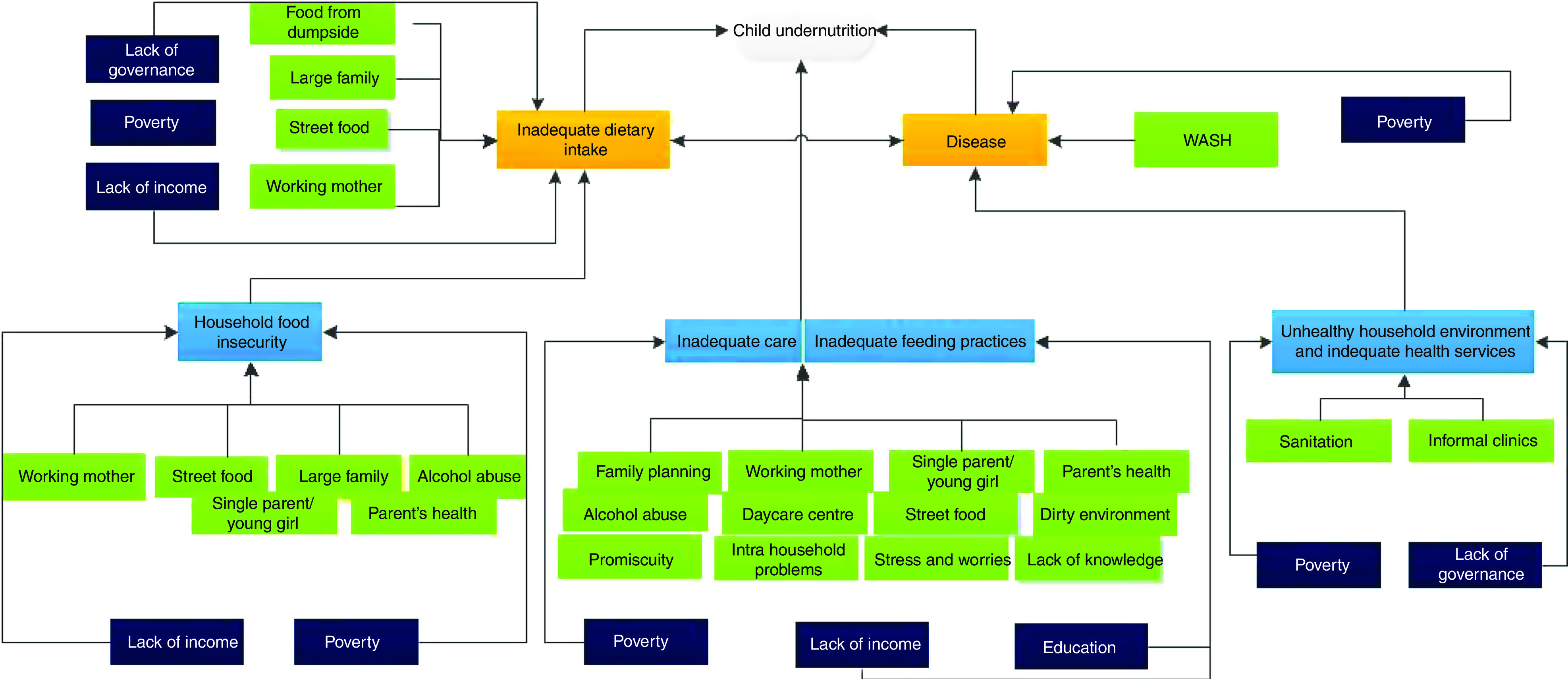
Conceptual framework of perceived causes of undernutrition in two slum communities in Nairobi, Kenya, mapped to the UNICEF conceptual framework (WASH, water, sanitation and hygiene)

The causes of undernutrition reported by participants were found to overlap in terms of broad categories with the UNICEF conceptual framework. However, the participants of both the FGD and the KII identified several more specific categories relevant to the urban slum setting. In Fig. 1, the UNICEF risk factors associated with undernutrition are presented in blue and orange and the findings from the participants in FGD and KII linked to each root cause are presented in green. The same risk factor may be associated with multiple root causes. For ease of understanding, these risk factors are mentioned in the ‘Results’ section under each cause. While all of the UNICEF immediate, underlying and basic causes of undernutrition were identified in participants’ narratives, we group and present here the results by the most important emerging themes based on participant responses: (i) maternal health and nutrition; (ii) IYCF practices; (iii) working mothers; (iv) family planning; (v) water, sanitation and hygiene (WASH) and related diseases; (vi) alcohol; and (vii) inadequate food and street food.

### Maternal health and nutrition

Mothers were described as unable or not willing to breast-feed as they themselves are undernourished. A young mother explained:

‘The mother eats one meal per day so if a baby breast-feeds too much then the mother also grows thin. So she stops the child early from breast-feeding.’ (FGD, young mothers, Korogocho)

A community elder added:

‘She hasn’t eaten. And if you look at the breasts, they are like *muratina* [a scrub sponge made from a plant product, which is thin and coarse]; they have fallen to the side.’ (FGD, community elders, Viwandani)

Young mothers reported that exclusive breast-feeding was not enough to promote their babies’ health due to their own poor nutritional status:

‘So even during those 6 months you are told to exclusively breast-feed, the child can get kwashiorkor because it sucks the food you are eating.’ (FGD, young mothers, Viwandani)

A young mother explained that maternal stress and worries and poor health can cause insufficient maternal milk production:

‘The baby just breast-feeds a little; the breasts have no milk then the mother starts saying she is feeling pain because the breasts have nothing. It’s the stress in your mind that causes the baby to have those problems.’ (FGD, young mothers, Viwandani)

### Suboptimal infant and young child feeding practices

Mothers are not well educated on child feeding, as a community health worker said:

‘The level of ignorance of mothers in the community is high and this really affects how these children grow.’ (FGD, community health workers, Viwandani)

Mothers were reported not to follow optimal breast-feeding practices and to introduce inappropriate complementary foods too early. Community health workers stated that babies at 1 month old were given ‘adult’ foods (FGD, community health workers, Korogocho). Young mothers were reported to refuse breast-feeding due to beauty reasons:

‘…as they claim the breasts will sag’ (FGD, community health workers, Viwandani)

Complementary foods are recommended by the WHO and by the Ministry of Health, Kenya to be introduced from 6 months of age, but suboptimal complementary foods were reported in the slums. These foods were introduced too early:

‘When the child cries it’s just given *mandazi* [a deep-fried snack made with wheat flour, water, sugar, and yeast or baking soda] and chips and that food cannot help the child.’ (FGD, community elders, Viwandani)

The complementary foods lack nutrients and the quantity is not right; ‘food does not satisfy them [children]’. An older mother stressed the lack of knowledge in nutrition:

‘Mothers in this community do not know the different foods to build, protect and add strength to the body; focus is on whether the child is full or not.’ (FGD, older mothers, Korogocho)

### Working mothers

In the slums, ‘people are not well off’ and as a health practitioner (KII, health professional, Viwandani) mentioned, ‘there is no money, so it’s hand to mouth’. In this context, mothers, often unemployed, have to look for a job, often in the informal sector. Maternal employment impacts on children’s dietary intake as children are not fed all day long when the mothers or caregivers are away in search for employment and revenue. Mothers’ permanent search for employment or their current employment means that they struggle to breast-feed. They are forced to leave their babies during the day and as a result they often discontinue breast-feeding. Maternal milk is then replaced with water, porridge or adult food. Employed mothers face limited options in terms of children’s care, leaving their children unattended, with neighbours or in a day-care centre. The day-care centres were described as overcrowded, with twenty children to one carer, often in one small room, and with unqualified staff to care for the children. When children are left unattended, they are left ‘in a dirty environment’ which brings sickness (FGD, young mothers, Korogocho). When babies are left at a day-care centre, foods given are milk mixed with water, or bananas. A community elder explained:

‘Most of these young mothers take children to school [day-care centre] and they are not given food, because they give a teacher a 6-month-old baby and they go to work and come back in the evening. That teacher will not have time to give the child food until it’s satisfied because the children are many. So the child stays hungry and when the teacher knows the mother is about to come is when she feeds it with the cold rice and the baby eats.’ (FGD, community elders, Viwandani)

### Family planning

The lack of access to good family planning services due to the presence of mainly ‘informal clinics’ was an important factor leading to the child’s poor health. A community health worker mentioned:

‘If a mother goes to a clinic in the community for family planning and in the process she gets pregnant, she will lose faith and will not take care of the child since in her mind she did not want another child; this child may even die due to neglect and poor health.’ (FGD, community health workers, Viwandani)

Participants explained that the absence of family planning is a risk factor:

‘You are not even able to take good care of the other older children yet you still get more. So those other children suffer.’ (FGD, young mothers, Korogocho)

Promiscuity was also cited as impacting on baby care, as noted strongly by a religious leader:

‘Blood is mixed. She has sexual relations with this one, then this one, tomorrow another one and she is a drunkard. She has no time to look after the baby.’ (KII, religious leader, Viwandani)

There were also cultural reasons. In some subgroups, women who are promiscuous are not expected to breast-feed their children because of ‘mixing of blood’, which would affect the baby.

Participants noted the issue of young adolescent girls having children and not taking responsibility of care for their children, which was also related to issues with family planning:

‘The young girls get pregnant too early and the men disappear and so they are left alone and so they don’t know how to bring up the child.’ (FGD, community health workers, Korogocho)

The responsibility of caring for the baby falls under the girl’s own mother (KII, traditional birth attendant, Korogocho) and as a women’s group leader reported, young girls themselves recognised the problem of neglecting their children:

‘We [young girls] don’t bother with children.’ (KII, women’s leader, Korogocho)

### Water, sanitation and hygiene and related disease

The slum is mainly considered ‘a bad and dirty environment’. Access to water and sanitation was reported by participants as ‘poor’. The presence of faeces due to open defecation, mud, open gutters/trenches and drinking dirty water were perceived as some of the causes of undernutrition and sickness. A religious leader reported that children are often:

‘… being left to play in mud, touching other people’s urine and faeces. You know here most people don’t have toilets so they use polythene bags and throw. They throw at night. It can even hit you.’ (KII, religious leader, Viwandani)

Participants also added that:

‘The children play in the gutters and the parents do not bother because of ignorance. So the children can easily be sick.’ (FGD, community health workers, Korogocho)

‘They are just those old torn cloths which are also dirty. If the parent comes from out there with muddy legs he just lies there and the baby is still there. The bed becomes very dirty.’ (KII, religious leader, Viwandani)

This exposure to mud is perceived to cause undernutrition.

Worms were reported to be common among children, believed to be caused by the dirty environment, and difficulties were reported in affording deworming drugs:

‘Another problem is that the children are not dewormed, they grow naturally. The environment is also bad. You see the mud they eat there, and such things. So when the child is born and there are worms in the stomach, if they cannot afford food, to afford the medicine for deworming is a problem.’ (FGD, community elders, Viwandani)

Diarrhoea was also noted to be common in children due to insufficient water and poor sanitation:

‘Also in the slums here, there is no water. Sanitation is another problem. So if they start giving the baby foods, you have seen our sewage flows through the middle of houses, leading to flies and everything … there is no adequate water to clean the baby’s utensils, there is no fuel. Because that little fuel she has bought is what she wants to use to cook for people’s food, so will not warm water for cleaning the baby’s utensils. So still diarrhoea cases will come in.’ (KII, health professional, Viwandani)

### Alcohol

Alcohol consumption was pointed out as a common problem among mothers with consequences on the child’s nutritional health, because when women are drunk they are unable to prepare food for their children. Alcohol consumption was expressed by participants as a major problem among residents of the slums, including mothers. A women’s group leader said:

‘I asked them one day why they drink [alcohol] like that, and they said they do it so as not to worry and it is true. She will sleep, while the child is crying outside. … If you walk through this community you can cry tears.’ (KII, women’s leader, Viwandani)

The alcohol consumed is illegal, cheap and very strong:

‘You find someone is drinking and has children and those children have no one to take care of them.’ (KII, religious leader, Viwandani)

‘Alcohol can also be given to children, that way unattended children sleep while the mother is away.’ (FGD, young mothers, Korogocho)

Relating to the vicious circle of unemployment and alcoholism, a religious leader explained:

‘One works for one week and then is idle the next week and when that person sits idle, they see there is no option because they sleep hungry, the child sleeps hungry and they wonder what to do. And when they see the baby crying because of hunger they feel sad and gets into alcoholism.’ (KII, religious leader, Viwandani)

Local governance was also criticised for contributing to the illegal alcohol business by letting it take place facilitated by briberies:

‘What contributes towards this too much alcoholism is the local administration we have in the neighbourhood because every Sunday they must be given money’. (KII, religious leader, Viwandani)

### Inadequate food and street food

Children in the slums do not eat enough food with the required nutrients. Children are reported to be fed once to twice daily: morning and/or evening. The middle meal is often skipped. Participants stated that mothers have so little money that they must make choices about which foods to give to their children:

‘Food has become expensive … If it is milk at KES 10 [equivalent to $US 0·10], the mother will prepare tea for all the children, or cook kales with a lot of water to make a meal because life is expensive.’ (FGD, community elders, Korogocho)

Either choice – a small amount of milky tea or a small amount of watery kale – is inadequate for nutritional needs.

Mothers also rely on street food to feed their young children, which was noted as detrimental to children’s health (Table 4). Meals lack quantity as well. In some households food is equally shared between the household members; the more children, the less food is allocated to each household member. Deprived from food during the day, hungry children search for food in dumpsites (referred to locally as *boma*; FGD, community elders, Viwandani) or in the gutters (FGD, community health workers, Korogocho; FGD, young mothers, Korogocho). Moreover, ‘the food is also not hygienic’, meaning it is not prepared and kept in a hygienic manner, hence it can cause sickness. Very often, the food given is street food sold by the roadside which is considered not appropriate for young children as it is not home cooked (refer to Table 4).

**Table 4:** Selected participant comments about street foods/foods sold at the roadside in focus group discussions (FGD) and key informant interviews (KII) conducted in two slum communities in Nairobi, Kenya, April 2012

‘The child is left around eating anything by the roadside and doesn’t get good nutrition.’ (FGD, young mothers, Korogocho)
‘You’ll find us mothers most of the time don’t care about cooking. Most of the time we give our children foods that are sold by the roadside.’ (KII, women’s group leader, Korogocho)
‘In my view, many people are jobless. Like me I have a baby, I just leave it there in the neighbourhood as I go looking for income, I buy food by the roadside which is not good for the baby.’ (FGD, young mothers, Korogocho)
‘The parent may not have money and so buy boiled rice at around KES 10 prepared beside the road. This meal may be taken for both lunch and supper to ensure the child does not sleep on an empty stomach.’ (FGD, community elders, Viwandani)

## Discussion

The current paper explored community members’ perceptions of the factors associated with undernutrition in urban poor settings in Nairobi, Kenya in relation to the UNICEF conceptual framework. Our study has shown that participants understand the linkages between root causes and child nutritional health as expressed in the UNICEF conceptual framework, which presents an important basis to foster change. The respondents’ narratives strongly linked infant and child undernutrition and inadequate dietary intake, household food insecurity, inappropriate care and feeding practices with risk factors that they identified. These risk factors are associated with immediate or underlying root causes and some are associated with both. We grouped water, sanitation and hygiene factors together into a ‘WASH’ factor. We also grouped having a large family, suboptimal family planning clinics and being a young mother together into a ‘family planning’ factor. The most reported risk factors were WASH and family planning, street food and working mothers, followed by being a single parent, alcohol abuse and maternal health. We focus our discussions on the emerging factors that are recurrently associated with root causes at various levels of the conceptual framework. This focus should assist the development of interventions to eliminate malnutrition.

The findings on risk factors are in line with a scoping review on risk factors statistically associated with children’s nutritional outcomes in slums, which had identified the same risk factors, but also add to the understanding of why these factors pose a risk to children’s nutritional status^(^ ^51^ ^)^. The narratives here provide contextual information to explore why, for example, poor maternal health can lead to poor nutritional outcomes in children. In the scoping review, mother’s education was the most reported risk factor, then the child’s age, the child’s gender, the child’s morbidity status, household income and family size. In relation to the WASH risk factor, a literature review on child urban malnutrition showed that the physical environment (including poor water and sanitation, pollution, open sewerage and contamination) was determined as one of the major risk factors for children’s health in urban settings^(^ ^52^ ^)^. Findings here also reinforce earlier evidence that unfavourable environments (including WASH and flood exposure) were considered by slum dwellers as one of the most important risk factors for undernutrition^(^ ^39^ ^)^.

### Policy recommendations

In a 2016 scoping review^(^ ^53^ ^)^, 29 % of interventions implemented in urban settings were unsuccessful in promoting desired nutritional outcomes in children in urban settings. The recommendations resulting from these were mostly that an intervention alone is not sufficient to tackle undernutrition. The root causes, also called demand-side factors, of undernutrition are multidimensional, as shown in the present study. Researchers as well as practitioners in the aid/development sector have recommended multisectoral approaches to effectively tackle the root causes of undernutrition^(^ ^54^ ^,^ ^55^ ^)^ and that interventions should target households, communities and societies to address the underlying societal determinants^(^ ^56^ ^,^ ^57^ ^)^.

### Use of targeting criteria at household and community level

The findings herein show that the risk factors are present at different levels and criteria can be applied at household and community levels to enable targeting of children at risk of undernutrition in urban areas. Individual anthropometric measurements of the child (weight-for-height and/or mid-upper arm circumference) are typically used in nutrition interventions to identify vulnerable children. In slums where millions of children and their caregivers live, relying on individual measurement means that all children need to be screened to identify the vulnerable ones, which is not feasible. Governments and development agencies working in urban settings need a methodology to identify where the most vulnerable children are now and are likely to be in the future. Vulnerability assessment at a higher level than the child level would be a useful undertaking. This will help actors to focus research and implementation on areas where needs are greatest. Factors such as having a large family, maternal employment or being a single parent could be included in household-level indicators. At the community level, communities lacking access to water, being close to a dumpsite, having extremely poor sanitation, lacking access to family planning or the presence of informal health facilities could all be criteria for identification of communities and children that are most likely to be at risk of undernutrition. This identification could be done via community-level surveys with the use of mapping to zone areas within slums that are more vulnerable. This would be less resource-intensive than using individual measures to identify the most vulnerable infants and young children. The provision of public health facilities common to the wealthy nations and the wealthy of low-income nations, such as treated water for drinking and cooking, flushing toilets connected to sanitary sewage disposal systems, safe and nutrient-dense foods, and sanitary food storage, would provide the greatest coverage. These measures would also come at the lowest long-term cost, as evidenced in the health and economic improvements of the high-income nations in the past century^(^ ^58^ ^)^.

### Interventions

The interventions proposed are based upon the risk factors identified and include: (i) improve maternal health and nutrition; (ii) promote knowledge towards optimal breast-feeding and complementary feeding; (iii) support mothers in their working roles with optimal day-care centres; (iv) increase access to family planning services; (v) improve WASH; (vi) tackle alcohol problems at all levels; and (vii) limit consumption of street foods through infant feeding counselling.

#### Improve maternal health and nutrition

From the narratives, mothers’ health and nutritional status was strongly associated with their children’s health. Mothers with improved nutritional health will be able to provide better care to their children, thus alleviating malnutrition and enhancing infant survival chances in the household. In slum settings, food-insecure women who are pregnant or breast-feeding should be targeted to receive food aid, to make them stronger and their babies healthier.

#### Promote knowledge towards optimal breast-feeding and complementary feeding

In the narratives, the lack of knowledge of young mothers was stressed by community health workers and older mothers. Poor feeding practices have been identified in Nairobi slums. Pilot projects such as the Baby-Friendly Community Initiative that aims to promote feeding practices to pregnant women are currently being reviewed in Kenya. The findings from this initiative showed that there was a significant increase in breast-feeding practice. Initial evaluation of such interventions in Nairobi slums is showing promising results for improving exclusive breast-feeding^(^ ^59^ ^)^.

#### Support mothers in their working roles with optimal day-care centres

Maternal work or search for work was identified in the narratives as posing a nutritional risk for infants. Previous studies showed that children of working mothers tend to have higher morbidity^(^ ^60^ ^)^ and poorer nutritional status^(^ ^61^ ^)^ in poor urban settings. Ndugwa and Zulu’s study^(^ ^62^ ^)^ in Nairobi slums showed that the effect of the mother’s work status may vary depending on the level of earnings, nature of the work and child-care arrangements while the woman is working. In this type of urban poor environment the need for income-generating activities is high, but a lack of adequate child care results in an increased risk of infant and young child malnutrition. Most of the existing literature has compared ‘well operated’ day-care centres, where supplementary foods and deworming are provided along with a low child/staff ratio (number of children per staff), *v*. ‘non-well operated’ day-care centres with a high child/staff ratio. Well-operated day-care centres with good-quality care had the potential to improve child nutritional outcomes in Nepal^(^ ^63^ ^)^ and in Brazil^(^ ^64^ ^)^. Our findings suggest that there is a need to recommend improvement of existing day-care centres into well-operated centres, for example by ensuring that there is regular inspection and provision of training by relevant government ministries. There is also a need to support breast-feeding mothers to allow them to work without discontinuing breast-feeding. Similar initiatives in Bangladesh are successful and mothers working in garment factories can express and hygienically store breast milk as well as breast-feed their children during lunch breaks^(^ ^65^ ^)^.

#### Increase access to family planning

Our findings on family planning are in line with a previous study reporting high levels of unintended pregnancy among the urban poor of Nairobi^(^ ^66^ ^)^. Unintended pregnancy adversely influences maternal and child health-seeking behaviours, birth outcomes and women’s quality of life^(^ ^67^ ^,^ ^68^ ^)^. In Nairobi slums, unintended pregnancies were identified previously to negatively impact breast-feeding and complementary feeding practices^(^ ^15^ ^)^. Unintended pregnancies in young adolescent girls as reported here not only compromise the child’s health outcomes but also the mother’s potential for education. Access to good-quality family planning should be promoted to couples to allow them to plan families and family planning promotion should also be encouraged to young adolescent girls with the support of the Kenyan ministries and their partnering organisations^(^ ^45^ ^)^.

#### Improve water, sanitation and hygiene

The environment was described as unhygienic by participants with indication of poor drainage systems, inadequate sanitation and uncollected garbage resulting in morbidity and undernutrition. Issues regarding WASH access and practices were found in other studies^(^ ^69^ ^–^ ^72^ ^)^. The vicious circle of diarrhoea and malnutrition is well established^(^ ^73^ ^)^ and unless improved sanitation and appropriate hygiene practices are provided in slums, children will keep on having diarrhoea and being undernourished^(^ ^71^ ^,^ ^72^ ^,^ ^74^ ^,^ ^75^ ^)^. Better sanitation and hygiene practices can be promoted at community level with the set up of sanitation committees ensuring that community latrines are cleaned, drains are unblocked, and that garbage is not thrown under people’s houses but gathered at a collection point (e.g. community-led total sanitation, CLTS). Rights to sanitation and water should also be promoted to slum-dwellers as often these communities are not part of the government national services scheme^(^ ^76^ ^)^.

#### Tackle alcohol problems at all levels

Alcohol consumption emerged as a recurrent problem underlying children’s poor nutritional health. It was identified as contributing to household insecurity and poor feeding and care practices. The problem with alcohol use is that it is associated with other risky behaviours and unsafe sex that can have consequences for infant and child health and nutrition. Kimani-Murage *et al*.^(^ ^37^ ^)^ presented alcoholism at individual, group and community level and showed its impact on breast-feeding practices^(^ ^37^ ^,^ ^77^ ^,^ ^78^ ^)^. Here we show that the impact of alcohol is perceived to extend beyond its negative influences on breast-feeding practices to impact on infants’ and young children’s nutritional outcomes at all levels. Community members report that problems with alcohol occur at all levels, from local governance being corrupted to allow illegal alcohol into the local market to individual parents developing problems with alcohol addiction. Government support to fight corruption in local communities as well as supporting alcoholic parents with their addictions will be important initiatives in this context.

#### Address street food consumption with infant feeding counselling

The results on street food consumption by infants confirm previous findings that the urban poor consume street foods on a regular basis because they are cheap and timesaving and can be bought in small quantities^(^ ^79^ ^–^ ^83^ ^)^, with the poorer community consuming street foods more frequently^(^ ^82^ ^)^. Available fast foods have the potential of meeting the energy needs for adolescent/adult health but may lack appropriate nutritional value^(^ ^82^ ^,^ ^83^ ^)^ and diversity^(^ ^84^ ^)^. Important here is the fact that street foods are being consumed by infants and young children. From the participants’ narratives, the foods often are inadequate due to poor nutrient density and safety. The fact that solid foods such as rice or chips are being fed to young infants is an indicator that more effort should be targeted to better counselling on appropriate foods for infant feeding, including addressing the issue of street food consumption. This should be done at the same time as improving food security in the household as often street foods remain the cheapest option.

### Strengths/limitations

The research strengths were that the participants defined the problem of child undernutrition in their community and that different categories of people were interviewed, making the findings more comprehensive. On the other hand, a limitation here is that while we analysed the data according to the UNICEF model of causes of malnutrition, we did not construct the full causal model with the participants during the data collection due to time constraints. Therefore, the factors raised by participants as causing malnutrition may not fully capture all that is in the model. For example, the political context did not come up adequately. In a previous publication we established the role of men (fathers or spouses) as key in IYCF^(^ ^17^ ^)^; however, we did not specifically involve fathers in the present study. Future studies in Nairobi need to specifically target father’s perceptions also.

## Conclusion

The present study addressed mothers’ (young and old), community elders’, community health workers’ and health-care professionals’ views on the causes of poor child nutritional outcomes. Our respondents recognised many of the factors leading to child undernutrition in urban poor settings. Their narratives provide a springboard for identification criteria at the household and community level of infants and young children vulnerable to undernutrition, as well as for interventions aimed at addressing child nutritional health. Respondents clearly showed awareness of the risk factors for poor child nutritional outcomes. Their narratives are critical messages for policy makers and reinforce the need to engage funding to tackle the risk factors of undernutrition associated with immediate and underlying root causes that were identified: WASH, family planning, maternal work, parents’ alcohol abuse and consumption of street foods. Findings suggest the focus of these interventions should be towards improvement of maternal health and nutrition, promotion of optimal breast-feeding and complementary feeding practices, increased access to family planning, supporting working mothers, tackling the issue of alcohol abuse, addressing street foods in more targeted infant feeding counselling, along with working to improve food security and the WASH situation in poor urban settings.
